# Assessing Preoperative (EORTC) QLQ-C30 Score in Elderly Patients with Colorectal Cancer: Results from a Prospective Cohort Study

**DOI:** 10.3390/jcm13206193

**Published:** 2024-10-17

**Authors:** Athina A. Samara, Alexandros Diamantis, Dimitrios Magouliotis, Maria Tolia, Vasileios Tsavalas, George Tzovaras, Konstantinos Tepetes

**Affiliations:** 1 Department of Surgery, University Hospital of Larissa, 41110 Larissa, Greece; alexandrosdoc@gmail.com (A.D.); dimitrios.magouliotis.18@ucl.ac.uk (D.M.); billtsav@gmail.com (V.T.); gtzovaras@hotmail.com (G.T.); tepetesk@gmail.com (K.T.); 2 Department of Radiotherapy, University of Crete, 73100 Chania, Greece; mariatolia1@gmail.com

**Keywords:** elderly, colorectal cancer, quality of life, preoperative assessment

## Abstract

**Background**: In the present study, we aimed to investigate the association between (EORTC) QLQ-C30 scores and both preoperative somatometric parameters and postoperative outcomes in elderly patients undergoing elective surgery for resectable colorectal cancer. **Methods**: The 118 elderly consecutive patients who underwent colorectal surgery for cancer in a single university’s surgical department between 01/2018 and 12/2018 were prospectively enrolled in the present study. All patients with an age > 65 years, diagnosed with resectable colorectal cancer, without metastatic disease, that underwent elective surgery were included prospectively in the present study. **Results**: Regarding patients’ characteristics, a negative correlation between preoperative QLQ (pQLQ) score and age (*p* = 0.001) and a positive correlation between body mass index (BMI) and pQLQ score (*p* = 0.048) were observed. Furthermore, there was a statistically significant difference (*p* = 0.004) in the mean pQLQ score between patients with rectal or colon cancer. Moreover, assessing pQLQ score was a useful tool in terms of postoperative recovery. Negative correlations between the pQLQ score and time (days) of beginning oral feeding (*p* < 0.001) and length of hospital stay (*p* = 0.004) were found. The pQLQ score was statistically significantly lower (*p* = 0.005) in patients who had any postoperative complication; however, there was no difference in patients with major complications. **Conclusions**: Advanced age, colon cancer and decreased BMI were negatively associated with preoperative QLQ. The assessment of pQLQ in elderly patients with colorectal cancer can be a useful predictive tool for postoperative complications, length of hospital stay and postoperative rehabilitation.

## 1. Introduction

Over the past two decades, there has been great scientific progress in the field of surgical oncology. Since the turn of the century, quality of life (QoL) in surgical patients has been considered sporadically throughout the literature as an outcome with secondary importance after mortality and morbidity [1,2]. Furthermore, the emergence of new chemotherapy drugs and innovative radiotherapy techniques, together with improved screening programs allowing for the diagnosis of cancer in earlier stages, has enhanced the prognosis of cancer patients and increased postoperative overall survival [3,4]. In line with these improvements, attention has been directed towards health-related quality of life (HR-QoL) as a key outcome measure in the assessment of cancer treatment efficacy [5].

Colorectal cancer (CRC) represents the third most commonly occurring cancer and the second leading cause of cancer-related deaths worldwide [6]. Surgical resection has a fundamental role in the management of patients with CRC; however, the overall postoperative complication rate can be as high as 30% [7]. In this context, the preoperative evaluation of health-related quality of life (HR-QoL), especially for elderly patients, should be an important component of the decision-making process [8]. The European Organization for Research and Treatment of Cancer (EORTC) QLQ-C30 questionnaire was developed to evaluate quality of life in patients with colorectal cancer [9].

Elderly individuals represent a special group of oncological patients, where the decision for the optimal treatment depends on comorbidities and patients’ personal preferences. Results from several studies report that older patients frequently prioritize functional outcomes, such as functional independence, more than survival [10,11]. Data regarding the preoperative functional status of elder oncological patients are expected to identify fit and frail patients and will optimize treatment for this patient group by modifying and individualizing the standard treatment due to a high risk of complications [11]. Elderly patients can undergo a major operation with similar outcomes to younger patients when careful selection and individualization is applied [12].

In the present study, we aimed to investigate the association between (EORTC) QLQ-C30 score and both preoperative somatometric parameters and postoperative outcomes in elderly patients undergoing elective surgery for resectable colorectal cancer.

## 2. Methods

### 2.1. Patients

A prospective study of 118 elderly patients who underwent colorectal surgery for cancer in a single university’s surgical department was conducted between 01/2018 and 12/2018. All patients with an age > 65 years, diagnosed with resectable colorectal cancer, without metastatic disease, that underwent elective surgery were included prospectively in the present study.

As exclusion criteria, the following were considered: age < 65 years old, an emergency operation, a non-resectable tumor, stage IV colorectal cancer and an ASA score (American Society of Anesthesiology) equal to IV. Preoperatively, socio-demographic data and medical history were collected. The pathological records of the included patients were prospectively recorded in a database in order to assess surgical margins.

### 2.2. Questionnaires and Scores

All patients were asked to answer a self-administered questionnaire of the European Organization for Research and Treatment of Cancer (EORTC) QLQ-C30 [9]. This questionnaire includes nine multi-item scales (five functional scales, three symptom scales and a global health and quality-of-life scale). The results of each score were estimated and recorded in a database with the respective patient’s medical and pathology records. All patients answered the Greek validated version of the questionnaire [13].

### 2.3. Ethical Considerations

The present study was designed in accordance with the declaration of Helsinki. Experimental therapeutic protocols are not applicable in this study, and all patients were treated with the gold-standard therapeutic management in accordance with the international guidelines. Moreover, all data were analyzed anonymously using code numbers with respect to the patient’s privacy and collected in the context of routine diagnostic and therapeutic procedures. Nevertheless, informed written consent was obtained from every patient enrolled in the present study. Approval of the ethical committee of the hospital was obtained.

### 2.4. Potential Bias Management

All consecutive patients diagnosed with a histopathology biopsy from a colonoscopy of colorectal cancer were included in the present study in order to avoid selection bias. Moreover, all patients were operated on by the same surgical team in order to eliminate confounders regarding the surgical techniques that were applied. All outcomes concerned the first 30 postoperative days and there were no missing data to interpret.

### 2.5. Statistical Analysis

Data were analyzed using SPSS v.25 (IBM Chicago, IL, USA). Normal distributions of the data were evaluated by application of the Shapiro–Wilk and Kolmogorov–Smirnov normality tests. Qualitative variables are presented as absolute (N) and relative (%) frequencies, and quantitative variables are presented as means with standard deviation (SD). Comparisons of the mean (EORTC) QLQ-C30 score between two groups were performed with the Mann–Whitney U-test for nonparametric data, as data were not following a normal distribution. Correlations of two quantitative variables were performed using the Spearman correlation test for nonparametric data, as data were not following a normal distribution. Logistic regression was used in order to identify independent risk factors. The significance level was set at 5% (0.05).

## 3. Results

A total of 118 patients who were diagnosed with colorectal cancer and underwent an R0 resection were recruited in the present study; 76 were male (64%) and 42 (36%) were female. The basic patients’ characteristics are displayed on Table 1. The median age of diagnosis was 73 years (range: 65–88). In 84 (71%) cases, the tumor was located in the colon, and in 34 (29%), in the rectum. In addition, 88 (75%) patients were classified as ASA I-II and 30 (25%) patients as ASA III. Regarding the stage of the disease, 41 (35%) patients had stage I CRC, 24 (20%) stage II disease and 53 (45%) stage III disease. In 72 (61%) patients, an open surgical approach was applied, and in 46 (39%), a minimally invasive one (laparoscopic).

Table 2 displays correlations between pQLQ score and quantitative variants. Regarding age, a negative correlation was observed between preoperative QLQ (pQLQ) score and age (*p* = 0.001). Increasing age was associated with a lower QLQ score and older patients had a deteriorated quality of life. In addition to this, a positive correlation (0.259) was found between preoperative body mass index (BMI) and QLQ score (*p* = 0.048), indicating that in colorectal cancer patients, a lower BMI is associated with a lower preoperative quality of life.

The mean QLQ score was 79.72% and 77.8% for male and female patients, respectively (*p* = 0.782). Furthermore, there was a statistically significant difference (*p* = 0.004) in the mean QLQ score between patients with rectal or colon cancer (85.45% and 76.44%, respectively). Regarding ASA (American Society of Anesthesiology) score, the mean QLQ score of patients classified as ASA I-II was 82.77% and 68.07% in ASA III patients (*p* < 0.001). When comparing the mean preoperative QLQ score in patients who were selected for an open or a laparoscopic operation (80.99% and 75.96% respectively), a statistically significant difference was not observed (*p* = 0.28).

Moreover, assessing pQLQ score was a useful tool in terms of postoperative recovery. Regarding time of postoperative oral feeding, a statistically significant (*p* < 0.001) negative correlation (−0.207) was found between pQLQ score and time (days) of beginning oral feeding. More specifically, patients with a better score had begun oral feeding earlier. In a similar manner, a statistically significant (*p* = 0.004) negative correlation (−0.286) between pQLQ score and length of hospital stay (days) was observed, indicating that patients with lower preoperative quality of life were in need of longer hospitalization.

In Table 3, statistical associations between pQLQ and bivariate values are reported. The mean pQLQ score of patients who had any postoperative complication was statistically significantly lower (*p* = 0.005) when compared with that of patients without any postoperative complications (75.03% and 84.49%, respectively). However, when comparing the mean pQLQ score in patients who had a major complication (79.22%) with that of patients where major complications were not presented (79.00%), the difference was not statistically different (*p* = 0.736). In logistic regression, only ASA score and postoperative complications were identified as independent contributors to preoperative quality of life.

Regarding the use of neoadjuvant chemo or radiotherapy, there was no statistically significant difference (*p* = 0.841) in pQLQ score between patients who had neoadjuvant chemo or radiotherapy (81.08%) and the group that had not (78.66%). Similarly, there was no statistically significant difference (*p* = 0.793) between patients who needed adjuvant chemo or radiotherapy (79.49%) compared with patients who were not in need of adjuvant therapy (78.38%).

Last but not least, in the multivariate analysis, in order to identify independent risk factors for postoperative major complications, only the preoperative (EORTC) QLQ-C30 score (*p* = 0.018) was identified as an independent predictive factor.

## 4. Discussion

Quality of life is a multidimensional, dynamic and subjective value that focuses on the patient’s perspective, including physical, functional, emotional and social well-being [14]. In this context, assessing QoL is an outcome of great importance in evaluating the full impact of a disease on patients and their families [15]. A variety of patient-reported outcome measures (PROMs) have been developed in order to assess patient-reported quality of life, including generic and disease-specific scales [16,17]. Generic PROMs are useful in comparing patients with different diseases (SF-36, EQ-5D), other ones are designed for oncological patients (EORTC QLQ-C30, FACT-G), and some are specific for colorectal cancer patients (EORTC-CR29, FACT-C) [17].

The resection of localized CRC remains the gold-standard method of treatment [18]. Surgery and its possible complications increase the risk of postoperative functional decline and loss of independence that are difficult to be regained [19]. The burden of treatments and their outcomes are considered as strong determinants of older patients’ preferences of treatment [20]. Geriatric patients typically report that the maintenance of independence and health-related quality of life represent major factors in treatment decision-making over standard oncological outcomes [21]. In general, surgery is considered as a safe option for older patients, and the primary risk factor for surgical complications or poor survival is not the age per se, but comorbidities, frailty and sarcopenia [21,22,23].

In the present study, (EORTC) QLQ-C30 score was preoperatively assessed in elderly patients >65 years old who underwent an elective operation for resectable colorectal cancer and associated with demographic and somatometric characteristics, tumor location, operative approach and parameters of the first 30-day postoperative period. Although recent studies have demonstrated an association between preoperative QLQ-C30 values and perioperative outcomes, the specific aspects of health-related quality of life that predict these outcomes remain unclear [24]. Law et al. have recently reported that the preoperative social function score, which reflects the impact of colorectal cancer treatment and diagnosis on the patient’s family and social life, is an independent predictor of postoperative readmission rates. Additionally, higher physical functioning scores were associated with a shorter length of hospital stay. While psychological distress and depression influence patients’ health-related quality of life, several studies have shown that psychological domains do not negatively affect postoperative outcomes [24,25].

Regarding demographical parameters, our findings conclude that patients > 65 years old had a statistically significantly decreased pQLQ; however, data from the literature are controversial [14]. In some studies, QoL increases with age [26], whereas others reported similar results with our findings [27,28]. Moreover, in line with the results from other studies [29], gender was not associated with a statistically significant difference in pQLQ. Nevertheless, other aspects of QoL such as sexual dysfunction seems to affect the male gender more than females [30]. A finding of interest is the fact that pQLQ does not seem to affect the decision to take an open or laparoscopic approach. Randomized controlled studies failed to find a statistically significant difference in terms of postoperative QoL for laparoscopic or open colorectal surgery; however, data from several studies have associated a laparoscopic approach with more favorable outcomes [31]. A finding of interest is the fact that according to our results, patients with rectal cancer report better pQLQ values compared to patients with colon cancer. These findings may be explained by the complex mechanical and physiological function of the gastrointestinal tract.

In our study, BMI was positively correlated with pQLQ, with a decreased BMI being associated with a decreased QoL. According to some authors, a possible survival advantage has been hypothesized in certain oncological settings in the presence of metastatic disease, raising the possibility that an ‘obesity paradox’ and a protective role of high BMI could exist for some cancer patients [32,33]. In addition, cancer anorexia–cachexia syndrome, a multifactorial clinical syndrome characterized by tissue wasting, loss of body weight, increased resting energy expenditure and metabolic alterations resulting in a decrease in BMI, has a negative impact on patients’ quality of life [34]. The correlation between BMI and QoL in CRC patients can be explained as site-specific, considering the crucial role of the colon and rectum in nutrition status. Moreover, neoadjuvant therapy may have an effect on BMI; however, information about BMI prior to neoadjuvant treatment were not available. Our population lacks extreme values of severely obese or severely underweight patients, and we cannot report values of pQLQ in these conditions.

In accordance with the results from previous studies, higher pQLQ values were associated with better postoperative recovery [35,36]. More specifically, a positive correlation was observed between pQLQ and early oral feeding. Oral feeding in colorectal cancer has a great impact on the patient’s nutrition status, wellbeing and self-satisfaction [37]. Moreover, patients with a better pQLQ are less likely to experience postoperative complications; however, here, patients that experienced a major complication did not have lower pQLQ scores. Our study group reported [8] our results regarding the predictive value of assessing pQLQ scores in elderly patients with CRC regarding postoperative complications. On the other hand, a lower pQLQ is associated with the need for a longer hospital stay. Several studies report that frail older patients were at risk of developing postoperative complications and had longer hospital stays [38,39,40].

Numerous explanations have been proposed to elucidate the consistent association between HRQoL and survival. Firstly, patient-reported HRQoL may provide a more accurate reflection of survival-related functioning and well-being compared to traditional prognostic indicators, such as clinician-reported performance status [41]. The EORTC summary score is composed of various questions with more sensitive response scales, capturing distinct and unique aspects of well-being [42]. Furthermore, the preoperative assessment of quality of life serves as a valuable predictive tool for post-treatment life expectancy across several cancer types [43,44].

Moreover, the preoperative assessment of GRADE score was strongly and significantly associated with 5-year postoperative mortality among older patients undergoing major oncological surgery [45]. The GRADE score requires only two minutes to assess and is an easy-to-perform daily surgical practice tool including cancer site and extension, weight loss and gait speed [45]. Future perspectives could include the correlation of GRADE score with quality of life scores in order to individualize geriatric oncological patients’ treatment.

The results of the present study underline the role of the preoperative assessment of EORT QLQ-C30 scores in elderly patients with malignancies located in the colon or rectum; however, before the appraisal of our findings, several limitations require careful acknowledgment. The present cohort included only elderly patients with resectable CRC; thus, the generalization of our results is limited for the general population. Moreover, as our study is one of the first ones to associate preoperative HRQoL scores with perioperative outcomes in colorectal cancer patients, the validation of our results by comparisons with the results of similar studies is not feasible. Future prospective well-designed studies are needed in order to validate our findings. On the other hand, the present study has several strengths, including no missing data on any QoL subscale for the entire study sample; a consistent, prospectively collected population of elderly patients older than 65 years with non-metastatic CRC who underwent elective surgery; the use of a valid and reliable QoL questionnaire; the availability of all clinical parameters in all individuals. Future perspectives of our study include the analysis of overall and disease-free survival data and their correlation with preoperative quality of life scores.

## 5. Conclusions

The assessment of health-related quality of life using (EORT) QLQ-C30 scores in elderly patients with malignancies located in the colon or rectum can be a useful predictive tool for assessing postoperative complications, the length of hospital stay and postoperative rehabilitation.

## Figures and Tables

**Table 1:** Patients’ basic characteristics.

Patients’ Characteristics	
Age	73.98
(mean, SD)	(6.25)
Male	76
(%)	(64%)
Female	42
(%)	(36)
BMI	24.76
(mean, SD)	(2.85)
ASA I-II	88
(%)	(75%)
ASA III	30
(%)	(25%)
Stage I	41
(%)	(35%)
Stage II	24
(%)	(20%)
Stage III	53
(%)	(45%)
Colon	84
(%)	(71%)
Rectum	34
(%)	(29%)
Open	72
(%)	(61%)
Laparoscopic	46
(%)	(39%)

**Table 2:** Correlations with quantitative variants.

Variant	Correlation	*p*-Value
Age	−0.436	0.001 *
BMI	0.259	0.048 *
Day of oral feeding (days)	0.207	<0.001 *
Length of hospitalization (days)	−0.286	0.004 *

* Spearman correlation test.

**Table 3:** Statistical associations with bivariate values.

Variant	Mean QLQ in the Groups	Univariate Analysis *p*-Value	Logistic Regression *p*-Value
Gender	Male: 79.72% Female: 77.8%	0.782 *	0.295
Location	Colon: 76.44% Rectum: 85.45%	0.04 *	0.194
ASA	I-II: 82.77% III: 68.07%	<0.001 *	0.002
Approach	Open: 80.99% Laparoscopic: 75.96%	0.28 *	0.303
Neoadjuvant therapy	Yes: 81.08% No: 78.66%	0.841 *	0.492
Any complication	Yes: 75.03% No: 84.49%	0.005 *	0.014
Major complication	Yes: 79.22% No: 79.00%	0.736 *	0.424
Adjuvant therapy	Yes: 79.49%, No: 78.38%	0.793 *	0.235

* Mann–Whitney test.

## Data Availability

Data is available upon request.

## References

[1] O’Boyle C.A. (1992). Assessment of quality of life in surgery. Br. J. Surg..

[2] Wyld L., Audisio R.A., Poston G.J. (2015). The evolution of cancer surgery and future perspectives. Nat. Rev. Clin. Oncol..

[3] Trimble E.L., Ungerleider R.S., Abrams J.A., Kaplan R.S., Feigal E.G., Smith M.A., Carter C.L., Friedman M.A. (1993). Neoadjuvant therapy in cancer treatment. Cancer.

[4] Versluis J.M., Thommen D.S., Blank C.U. (2020). Rationalizing the pathway to personalized neoadjuvant immunotherapy: The Lombard Street Approach. J. Immunother. Cancer.

[5] Kaal S.E.J., Lidington E.K., Prins J.B., Jansen R., Manten-Horst E., Servaes P., van der Graaf W.T., Husson O. (2021). Health-Related Quality of Life Issues in Adolescents and Young Adults with Cancer: Discrepancies with the Perceptions of Health Care Professionals. J. Clin. Med..

[6] Wang X.Y., Zhang R., Wang Z., Geng Y., Lin J., Ma K., Zuo J.L., Lu L., Zhang J.B., Zhu W.W. (2019). Meta-analysis of the association between primary tumour location and prognosis after surgical resection of colorectal liver metastases. Br. J. Surg..

[7] Brown S.R., Mathew R., Keding A., Marshall H.C., Brown J.M., Jayne D.G. (2014). The impact of postoperative complications on long-term quality of life after curative colorectal cancer surgery. Ann. Surg..

[8] Diamantis A., Samara A.A., Tzovaras G., Magouliotis D., Arnaoutoglou E., Volakakis G., Tepetes K. (2021). Use of preoperative EORTC quality-of-life questionnaires to predict postoperative complications after colorectal cancer surgery. Br. J. Surg..

[9] Aaronson N.K., Ahmedzai S., Bergman B., Bullinger M., Cull A., Duez N.J., Filiberti A., Flechtner H., Fleishman S.B., de Haes J.C. (1993). The European Organization for Research and Treatment of Cancer QLQ-C30: A quality-of-life instrument for use in international clinical trials in oncology. J. Natl. Cancer Inst..

[10] Akishita M., Ishii S., Kojima T., Kozaki K., Kuzuya M., Arai H., Arai H., Eto M., Takahashi R., Endo H. (2013). Priorities of health care outcomes for the elderly. J. Am. Med. Dir. Assoc..

[11] Rønning B., Wyller T.B., Nesbakken A., Skovlund E., Jordhøy M.S., Bakka A., Rostoft S. (2016). Quality of life in older and frail patients after surgery for colorectal cancer-A follow-up study. J. Geriatr. Oncol..

[12] Simmonds P.D., Best L., George S., Baughan C., Buchanan R., Davis C., Fentiman I., Gosney M., Northover J., Williams C. (2000). Surgery for colorectal cancer in elderly patients: A systematic review. Lancet.

[13] Kyriaki M., Eleni T., Efi P., Ourania K., Vassilios S., Lambros V. (2001). The EORTC core quality of life questionnaire (QLQ-C30, version 3.0) in terminally ill cancer patients under palliative care: Validity and reliability in a Hellenic sample. Int. J. Cancer.

[14] Marventano S., Forjaz M., Grosso G., Mistretta A., Giorgianni G., Platania A., Gangi S., Basile F., Biondi A. (2013). Health related quality of life in colorectal cancer patients: State of the art. BMC Surg..

[15] Sahay T.B., Gray R.E., Fitch M. (2000). A qualitative study of patient perspectives on colorectal cancer. Cancer Pract..

[16] Pachler J., Wille-Jørgensen P. (2012). Quality of life after rectal resection for cancer, with or without permanent colostomy. Cochrane Database Syst. Rev..

[17] McNair A.G., Whistance R.N., Forsythe R.O., Rees J., Jones J.E., Pullyblank A.M., Avery K.N., Brookes S.T., Thomas M.G., Sylvester P.A. (2015). Synthesis and summary of patient-reported outcome measures to inform the development of a core outcome set in colorectal cancer surgery. Color. Dis..

[18] Symeonidis D., Tepetes K., Tzovaras G., Kissa L., Samara A.A., Bompou E., Zacharoulis D. (2022). Colorectal Cancer Liver Metastases: Is an R1 Hepatic Resection Accepted?. Clin. Pract..

[19] Lawrence V.A., Hazuda H.P., Cornell J.E., Pederson T., Bradshaw P.T., Mulrow C.D., Page C.P. (2004). Functional independence after major abdominal surgery in the elderly. J. Am. Coll. Surg..

[20] Fried T.R., Bradley E.H., Towle V.R., Allore H. (2002). Understanding the treatment preferences of seriously ill patients. N. Engl. J. Med..

[21] Dolin T.G., Mikkelsen M., Jakobsen H.L., Nordentoft T., Pedersen T.S., Vinther A., Zerahn B., Vistisen K.K., Suetta C., Nielsen D. (2021). Geriatric assessment and intervention in older vulnerable patients undergoing surgery for colorectal cancer: A protocol for a randomised controlled trial (GEPOC trial). BMC Geriatr..

[22] Simonsen C., de Heer P., Bjerre E.D., Suetta C., Hojman P., Pedersen B.K., Svendsen L.B., Christensen J.F. (2018). Sarcopenia and Postoperative Complication Risk in Gastrointestinal Surgical Oncology: A Meta-analysis. Ann. Surg..

[23] Ommundsen N., Wyller T.B., Nesbakken A., Jordhøy M.S., Bakka A., Skovlund E., Rostoft S. (2014). Frailty is an independent predictor of survival in older patients with colorectal cancer. Oncologist.

[24] Law J.H., Lau J., Pang N.Q., Khoo A.M., Cheong W.K., Lieske B., Chong C.S., Lee K.C., Tan I.J., Siew B.E. (2023). Preoperative Quality of Life and Mental Health Can Predict Postoperative Outcomes and Quality of Life after Colorectal Cancer Surgery. Medicina.

[25] Geoffrion R., Koenig N.A., Zheng M., Sinclair N., Brotto L.A., Lee T., Larouche M. (2021). Preoperative Depression and Anxiety Impact on Inpatient Surgery Outcomes: A Prospective Cohort Study. Ann. Surg. Open.

[26] Hamashima C. (2002). Long-term quality of life of postoperative rectal cancer patients. J. Gastroenterol. Hepatol..

[27] Sapp A.L., Trentham-Dietz A., Newcomb P.A., Hampton J.M., Moinpour C.M., Remington P.L. (2003). Social networks and quality of life among female long-term colorectal cancer survivors. Cancer.

[28] Trentham-Dietz A., Remington P.L., Moinpour C.M., Hampton J.M., Sapp A.L., Newcomb P.A. (2003). Health-related quality of life in female long-term colorectal cancer survivors. Oncologist.

[29] Dibble S.L., Padilla G.V., Dodd M.J., Miaskowski C. (1998). Gender differences in the dimensions of quality of life. Oncol. Nurs. Forum.

[30] Samara A.A., Baloyiannis I., Perivoliotis K., Symeonidis D., Diamantis A., Tepetes K. (2021). Intraoperative neuromonitoring in rectal cancer surgery: A systematic review and meta-analysis. Int. J. Color. Dis..

[31] Bartels S.A., Vlug M.S., Ubbink D.T., Bemelman W.A. (2010). Quality of life after laparoscopic and open colorectal surgery: A systematic review. World J. Gastroenterol..

[32] Formica V., Nardecchia A., Morelli C., Lucchetti J., Giuliano G., Renzi N., Gallo C., Pellegrino R., Massimiliani V., Serci C. (2021). Health-related quality of life in patients with advanced colorectal cancer: A predictive nomogram including BMI, sex and age. Ann. Palliat. Med..

[33] Pamoukdjian F., Aparicio T., Canoui-Poitrine F., Duchemann B., Lévy V., Wind P., Ganne N., Sebbane G., Zelek L., Paillaud E. (2019). Obesity survival paradox in cancer patients: Results from the Physical Frailty in older adult cancer patients (PF-EC) study. Clin. Nutr..

[34] Tarricone R., Ricca G., Nyanzi-Wakholi B., Medina-Lara A. (2016). Impact of cancer anorexia-cachexia syndrome on health-related quality of life and resource utilisation: A systematic review. Crit. Rev. Oncol. Hematol..

[35] Nilsson U., Dahlberg K., Jaensson M. (2019). Low Preoperative Mental and Physical Health is Associated with Poorer Postoperative Recovery in Patients Undergoing Day Surgery: A Secondary Analysis from a Randomized Controlled Study. World J. Surg..

[36] Hälleberg Nyman M., Nilsson U., Dahlberg K., Jaensson M. (2018). Association Between Functional Health Literacy and Postoperative Recovery, Health Care Contacts, and Health-Related Quality of Life Among Patients Undergoing Day Surgery: Secondary Analysis of a Randomized Clinical Trial. JAMA Surg..

[37] Fernández-Martínez D., Rodríguez-Infante A., Otero-Díez J.L., Baldonedo-Cernuda R.F., Mosteiro-Díaz M.P., García-Flórez L.J. (2020). Is my life going to change?—A review of quality of life after rectal resection. J. Gastrointest. Oncol..

[38] Fagard K., Leonard S., Deschodt M., Devriendt E., Wolthuis A., Prenen H., Flamaing J., Milisen K., Wildiers H., Kenis C. (2016). The impact of frailty on postoperative outcomes in individuals aged 65 and over undergoing elective surgery for colorectal cancer: A systematic review. J. Geriatr. Oncol..

[39] Saxton A., Velanovich V. (2011). Preoperative frailty and quality of life as predictors of postoperative complications. Ann. Surg..

[40] Cai M., Gao Z., Liao J., Jiang Y., He Y. (2022). Frailty affects prognosis in patients with colorectal cancer: A systematic review and meta-analysis. Front. Oncol..

[41] Gotay C.C., Kawamoto C.T., Bottomley A., Efficace F. (2008). The prognostic significance of patient-reported outcomes in cancer clinical trials. J. Clin. Oncol..

[42] Husson O., de Rooij B.H., Kieffer J., Oerlemans S., Mols F., Aaronson N.K., van der Graaf W.T.A., van de Poll-Franse L.V. (2020). The EORTC QLQ-C30 Summary Score as Prognostic Factor for Survival of Patients with Cancer in the “Real-World”: Results from the Population-Based PROFILES Registry. Oncologist.

[43] Inoue J., Morishita S., Okayama T., Suzuki K., Tanaka T., Nakano J., Fukushima T. (2024). Impact of quality of life on mortality risk in patients with esophageal cancer: A systematic review and meta-analysis. Esophagus.

[44] Fukushima T., Suzuki K., Tanaka T., Okayama T., Inoue J., Morishita S., Nakano J. (2024). Global quality of life and mortality risk in patients with cancer: A systematic review and meta-analysis. Qual. Life Res..

[45] Wind P., Ap Thomas Z., Laurent M., Aparicio T., Siebert M., Audureau E., Paillaud E., Bousquet G., Pamoukdjian F. (2021). The Pre-Operative GRADE Score Is Associated with 5-Year Survival among Older Patients with Cancer Undergoing Surgery. Cancers.

